# Low levels of respiratory syncytial virus activity in Europe during the 2020/21 season: what can we expect in the coming summer and autumn/winter?

**DOI:** 10.2807/1560-7917.ES.2021.26.29.2100639

**Published:** 2021-07-22

**Authors:** Jojanneke van Summeren, Adam Meijer, Guðrún Aspelund, Jean Sebastien Casalegno, Guðrún Erna, Uy Hoang, Bruno Lina, VRS study group in Lyon, Simon de Lusignan, Anne C. Teirlinck, Valtýr Thors, John Paget, VRS study group in Lyon, VRS study group in Lyon, Antoine Ouziel, Jean-claude Tardy, Pascal Gaucherand, Jerome Massardier, Stephanie Polazzi, Antoine Duclos, Mehdi Benchaib, Regine Cartier, Marine Jourdain, Michelle Ottmann, Rolf Kramer, Sylvie Fiorini, Nathalie Rivat, Yahia Mekki, Julie Fort-Jacquier, Maud-Catherine Barral, Vey Noelie, Julie Haesebaert, Come Horvat, Leo Vidoni, Jean-Marc Reynes, Jean-Francois Eleouet, Laurence Josset, Matthieu Receveur, Etienne Javouhey, Dominique Ploin, Martine Valette, Remi Fanget, Sandrine Couray Targe, Anne-Florence Myar-Dury, Muriel Doret-Dion, Mona Massoud, Elsa Masson, Emilie Bard, Gregory Queromes, Phillipe Vanhems, Olivier Claris, Marine Butin, Florence Ader, Sylvie Bin, Alexandre Gaymard, Florence Morfin, Yves Gillet

**Affiliations:** 1 Nivel, Netherlands Institute for Health Services Research, Utrecht, the Netherlands; 2 Centre for Infectious Diseases Research, Diagnostics and laboratory Surveillance, National Institute for Public Health and the Environment (RIVM), Bilthoven, the Netherlands; 3 Centre for Health Security and Communicable Disease Control, The Directorate of Health, Reykjavik, Iceland; 4 Virology Department, Institut des Agents Infectieux, Hôpital de la Croix-Rousse, HCL, Lyon, France; 5 Department of Clinical Microbiology, Landspitali University Hospital, Reykjavik, Iceland; 6 Oxford-Royal College of General Practitioners Research and Surveillance Centre, Nuffield Department of Primary Care Health Sciences, University of Oxford, Oxford, United Kingdom; 7 The members of the group are listed under Investigators; 8 Centre for Infectious Diseases, Epidemiology and Surveillance, National Institute for Public Health and the Environment (RIVM), Bilthoven, the Netherlands; 9 Children’s Hospital, Reykjavik, Iceland; 10 Faculty of Medicine, University of Iceland, Reykjavik, Iceland

**Keywords:** Respiratory syncytial virus, RSV, COVID-19 pandemic, epidemiology, surveillance data

## Abstract

Since the introduction of non-pharmacological interventions to control COVID-19, respiratory syncytial virus (RSV) activity in Europe has been limited. Surveillance data for 17 countries showed delayed RSV epidemics in France (≥ 12 w) and Iceland (≥ 4 w) during the 2020/21 season. RSV cases (predominantly small children) in France and Iceland were older compared with previous seasons. We hypothesise that future RSV epidemic(s) could start outside the usual autumn/winter season and be larger than expected. Year-round surveillance of RSV is of critical importance.

In December 2019, the novel severe acute respiratory syndrome coronavirus 2 (SARS-CoV-2) causing coronavirus disease (COVID-19), started circulating in Wuhan, China. The outbreak rapidly evolved and on 11 March 2020, the World Health Organization (WHO) declared the COVID-19 pandemic [1]. The number of patients hospitalised with COVID-19 increased quickly in Europe, and strict non-pharmacological interventions (NPIs) including lockdowns were applied. The set of NPIs that were applied differed across European countries and over time. The NPIs were not only effective in reducing the spread of the SARS-CoV-2 virus, but also led to a decline in most seasonal respiratory viruses, including respiratory syncytial virus (RSV) [2].

## Low levels of respiratory syncytial virus activity in Europe since the start of the COVID-19 pandemic

**Figure 1:**
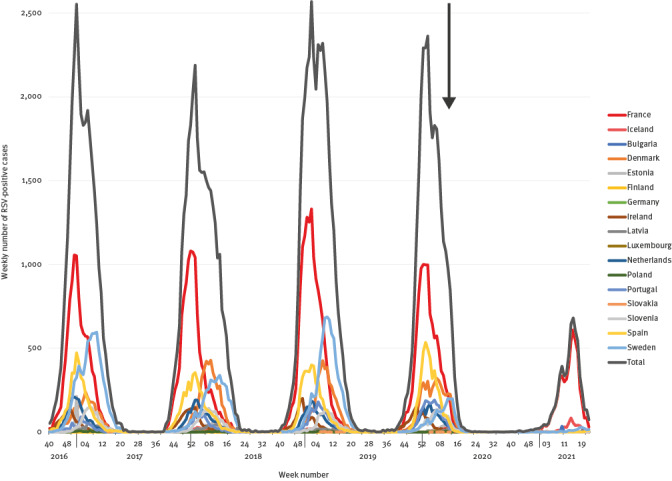
Respiratory syncytial virus activity in Europe, week 40 2016 to week 20 2021 (n = 17 European Union countries)

RSV: respiratory syncytial virus; TESSy: the European Surveillance System.

In the majority of the 17 European countries for which data using the European Centre for Disease Prevention and Control (ECDC) surveillance atlas data (https://www.ecdc.europa.eu/en/surveillance-atlas-infectious-diseases) is available (16 of the 27 European Union countries and Iceland), the circulation of RSV stopped immediately after NPIs were introduced to control SARS-CoV-2 circulation in February–March 2020 [3,4]. Since then, RSV epidemics have only been observed in France and Iceland during the 2020/21 winter, with epidemics starting several weeks later than usual (Figure 1). Since mid-May 2021, RSV has started to circulate in a number of countries, e.g. Sweden, the Netherlands, Spain, Portugal and Denmark, but with only small numbers up to week 20 (no later data available).

The 17 countries represented were selected based on data completeness. The ‘total’ line represents the weekly number of RSV-positive cases in the 17 countries. Data were extracted via the surveillance atlas, which uses the infrastructure of TESSy by the European Centre for Disease Prevention and Control and the World Health Organization. The TESSy surveillance network is described in detail elsewhere [32]. RSV positive-cases in week 53 2020/21 were added to week 52 2020/21. The black arrow represents the declaration of the COVID-19 pandemic.

**Figure 2:**
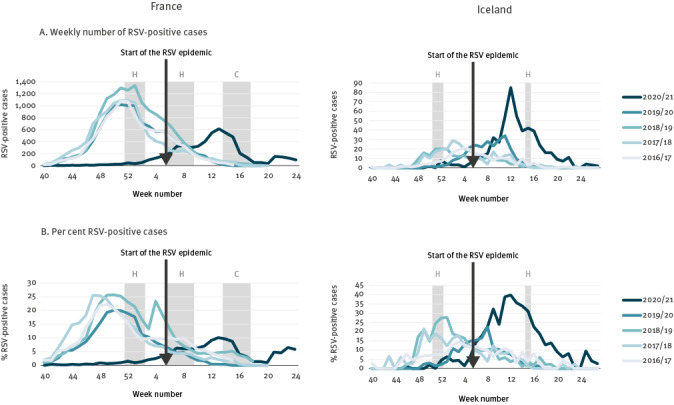
Respiratory syncytial virus activity in France, week 40 2016−week 24 2021 and Iceland, week 40 2016−week 27 2021

COVID-19: coronavirus disease; RSV: respiratory syncytial virus.

In France, the RSV epidemic started in week 5 2021, while in the seasons 2016/17 to 2019/20, the RSV epidemics started between weeks 44 and 46, which reveals a delayed start of at least 12 weeks (Figure 2). Moreover, the epidemic in 2020/21 lasted only 12 weeks compared with 14-15 weeks for previous seasons. In addition, the size of the peak was about half of that of the previous seasons, although there was no difference in the mean number of tests taken per week [5]. In Iceland, the RSV epidemic started in week 6 and ended in week 19, while in the seasons 2016/17 to 2019/20, the start was between week 48 and week 2 of the following year and the end between week 9 and 12 (Figure 2). In contrast to France, the season was similar in length compared with previous seasons, 13 weeks vs 11–13 weeks, respectively, but the size of the peak was four-fold higher compared with previous seasons; this greater peak size can be partially explained by a 21% increase in testing in the 2020/21 season. Importantly, RSV-related hospital admissions in Iceland were about half the number of previous seasons. We also examined the percentage of positive cases between seasons in France and Iceland and found that the patterns were similar to the number of cases (Figure 2). 

Weekly number and per cent of RSV-positive cases and school closures due to holidays (H) or COVID-19 restrictions (C). In France, the duration of the holiday in February–March was 2 weeks with a shift over three regions. National RSV surveillance in France has been stopped since week 24/2021. RSV-positive cases in week 53 2020/21 were added to week 52 2020/21.

Data source: Sentinel and non-sentinel surveillance data was collected via the infrastructure of The European Influenza Surveillance Network (France and Iceland).

**Figure 3:**
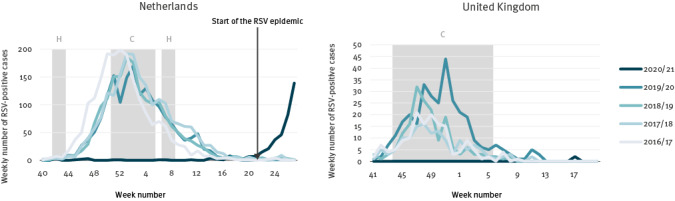
Respiratory syncytial virus activity in the Netherlands, week 40 2016-week 27 2021 and the United Kingdom, week 41 2016-week 20 2021

COVID-19: coronavirus disease; RSV: respiratory syncytial virus; UK: United Kingdom.

In the United Kingdom (UK) (data not included in the ECDC surveillance atlas) and the Netherlands, both members of the RSV Community Network (RSV ComNet), there was no RSV epidemic in the 2020/21 winter season. However, in the Netherlands, a very late out-of-season RSV epidemic started in week 24 2021, compared with a start between weeks 46 and 48 in previous seasons, which is a delay of at least 28 weeks (Figure 3). As at publication, hardly any RSV activity has been observed in the UK, but a small increase in RSV cases was detected in the last week (week 28) via the English sentinel surveillance system.

Weekly number of RSV-positive cases and school closures due to holidays (H) or COVID-19 restrictions (C). In the Netherlands, numbers in week 26 and 27 could be higher because not all laboratories have updated the numbers. Also, in the Netherlands, the duration of both holidays was 1 week with a shift over three regions. RSV-positive cases in week 53 2020/21 were added to week 52 2020/21.

Data source: Virological data from the weekly non-sentinel surveillance system in the Netherlands (NWKV) and the English sentinel surveillance system (Oxford-Royal College of General Practitioners Research and Surveillance Centre).

## Increase in the age of infected children in the 2020/21 season

We accessed additional data from Lyon, the second largest city in France, from a birth cohort that included infants (< 1 year of age) admitted to the hospital with respiratory symptoms and who tested positive for RSV [6], also co-published in this issue. The median age of children was increased compared with previous seasons: 4.8 months in 2020/21 compared with 2.2 to 3.1 months in the seasons 2016/17–2019/20. Similarly, in Iceland, the median age of RSV-positive cases in the five previous RSV seasons was 5.7 months (range: 3.2–6.1), but in the 2020/21 season it was 16.0 months, with a threefold increase in 1–2-year-olds. This age increase was also observed in Australia, where the median age of children hospitalised or visiting an emergency department with RSV in Western Australia was significantly higher (18.4 months) in 2020 compared with the years 2012 to 2019 (7.3–12.5 months), and in the State of Victoria the median age of hospitalised children with RSV was 13.2 months in 2020 compared with 11.4 months in 2017 to 2019 [7,8].

## Respiratory syncytial virus activity in the southern hemisphere during the COVID-19 pandemic

The absence of RSV circulation was also observed in countries with temperate climates in the southern hemisphere, such as Australia, Argentina, Chile, New Zealand, and South Africa. In 2020, hardly any RSV was detected in these countries during the southern hemisphere winter (June to September) [7,9-13]. In the first weeks of 2020, RSV activity was comparable to previous years, but after the introduction of NPIs (early April 2020), RSV activity disappeared [7,9]. In Australia and South Africa, RSV started to circulate at the beginning of spring (mid- to late August 2020), the period in which the RSV epidemic usually ends. In Chile and Argentina, where strict NPIs were maintained, there was hardly any RSV activity observed in the surveillance data since the start of the pandemic, but since April 2021, RSV activity is increasing in Argentina [11].

The duration of the RSV season in Australia and South Africa was also shorter compared with previous seasons [12,14]. In New South Wales (NSW), the 2020 RSV season lasted 4 months, compared with an average of 6 months in 2016 to 2019 [12]. Since mid-January, the sharp decline in the number of RSV positive cases has levelled off in NSW, and since mid-May the numbers are increasing to the levels similar to usual winter periods. This means that the late RSV epidemic has moved up into the usual winter period [12]. In South Africa, the delayed RSV epidemic lasted only 5 weeks compared with 19 to 33 weeks in 2009 to 2016 [14,15]. In 2021 (data available to week 25), the RSV activity remained below seasonal thresholds [14].

## Impact of non-pharmacological interventions 

The COVID-19 pandemic shows that NPIs have a large preventive impact on the transmission of RSV. However, it is difficult to measure the impact of each NPI separately because, while there was always a set of measures applied during the COVID-19 pandemic, the measures changed over time. In France and Iceland, the NPIs were gradually relaxed from November 2020 to February 2021, while the measures became stricter over time in the Netherlands and the UK [16].

In New South Wales and Western Australia, normal life had largely resumed since June 2020 (with the exception of closed borders) before RSV activity was observed since mid-August [8,9,12]. In the state of Victoria, strict NPIs were applied for a longer period and the RSV epidemic started 4 months later compared with the other two states [8,9]. The difference in the size of the RSV epidemics might also be explained by the stringency of NPIs; in Australia most of the NPIs were lifted while in South Africa, the NPIs were relaxed but are still in effect today [16].

It might be hypothesised that primary school and day care facilities closures have an important impact on RSV transmission, as RSV is predominantly detected in young children [17]. In countries without an RSV epidemic, primary schools and day care centres were closed due to COVID-19 restrictions between November and March for at least 8 weeks (the Netherlands) and 9 weeks (UK) (Figure 3). On the contrary, in France and Iceland, primary schools and day care facilities were not closed, but some additional restrictions in schools were applied (Figure 2). Australia and South Africa also did not have primary school closures due to COVID-19 restrictions in the weeks before their RSV epidemics started [7,10,16]. We also found that a decline in the number of RSV-positive cases in France seems to have been observed after the closure of schools due to holidays and COVID-19 restrictions in April (Figure 2). This supports our finding that school closures have an impact on RSV epidemics.

## Ethical statement

Ethical approval was not required because this study uses routine surveillance data for which no patient consent is required.

## Discussion

RSV epidemics were only observed in Europe during the 2020/21 season in France and Iceland, countries that had a policy of keeping their primary schools and day care facilities open. In the Netherlands, the RSV epidemic started 19 weeks after schools were reopened, suggesting that school closures had an impact on RSV activity. The impact of school closures has also been established in other directly transmitted infectious diseases such as measles and influenza [18-20]. The COVID-19 stringency index, developed by the Oxford COVID-19 Government Response Tracker group [16], suggests that more stringent measures were applied in countries without an RSV epidemic compared with the countries with an RSV epidemic, but further research is needed to confirm these findings and to evaluate the impact of individual NPIs on RSV activity.

The timing of the next RSV epidemic might be associated with a relaxation of international travel restrictions [21,22]. An increase in travelling to countries with RSV activity may facilitate the spread of RSV to other European countries [22]. Other factors which are believed to influence the epidemiology of respiratory viruses such as temperature, humidity and crowding in school classrooms may also influence the chances of future RSV epidemics during an unusual time of the year [21,23,24]. In addition, the RSV epidemic was also delayed during the 2009 influenza pandemic, which further suggests evidence for viral interference; the dominance of the SARS-CoV-2 virus might also prevent the activity of RSV [25].

The increased age of RSV infected children in countries with an RSV epidemic since the start of the COVID-19 pandemic is noteworthy. Normally, 60–70% of infants under the age of 1 year and almost all children under the age of 2 years will develop an RSV infection at some point [26,27]. These children subsequently develop immunity that protects them against severe RSV infection [27-29]. Due to the absence of RSV activity since the start of the COVID-19 pandemic, a larger number of infants and young children (with a slight upward age shift) may be at an increased risk for severe RSV infection because they did not have had the opportunity to develop immunity against severe infection earlier [28-30]. It is unclear whether children with a first RSV infection at an older age are less prone to a severe infection. Clinicians therefore need to be prepared for the next RSV epidemic(s) that include older children, and possibly with cohorts of a larger size.

Existing passive immunisation policies for high-risk infants might have to be reconsidered with respect to (i) extending the indications of passive immunisation to slightly older infants, (ii) administering the passive immunisation outside the usual winter season and (iii) administering the passive immunisation for a longer period of time.

RSV is currently not an official notifiable infectious disease that is monitored by the ECDC. In most European countries, RSV surveillance is part of the influenza surveillance network and is reported to ECDC, although this is not the case in all European countries. This hampers the ability to get a representative picture of RSV activity in Europe (e.g. Belgium observed a late RSV epidemic in the 2020/21, which we only became aware of after our analyses were completed) [31]. National and collective European surveillance of RSV is important as RSV infections in small children have a considerable burden on healthcare systems in Europe.
