# Authors’ correction for Euro Surveill. 2021;26(29)

**DOI:** 10.2807/1560-7917.ES.2021.26.30.210729c1

**Published:** 2021-07-29

**Authors:** Eurosurveillance editorial team

**Affiliations:** 1 European Centre for Disease Prevention and Control (ECDC), Stockholm, Sweden

On request of the authors, the sentence “JvS, AM, UH, SdL, and JP were contracted on a Sanofi Pasteur and AstraZeneca funded research project ‘RSV ComNet’ to measure the burden of RSV in young children in primary care during manuscript development” was changed to “JvS, AM, UH, SdeL, and JP report that this manuscript was prepared through their participation in the RSV ComNet project, which is a research project that is funded by Sanofi Pasteur and AstraZeneca” and all instances of Simon de Lusignan’s name abbreviation were changed from “SdL” to “SdeL” in the article ‘Low levels of respiratory syncytial virus activity in Europe during the 2020/21 season: what can we expect in the coming summer and autumn/winter?’ by van Summeren et al., published on 22 July 2021. These changes were made on 29 July 2021. 

